# Impact of electromagnetic stimulation on the mechanical and photophysical properties of alfalfa leaves

**DOI:** 10.1038/s41598-022-20737-z

**Published:** 2022-10-06

**Authors:** Agata Dziwulska-Hunek, Mariusz Szymanek, Arkadiusz Matwijczuk, Norbert Leszczyński, Agnieszka Niemczynowicz, Beata Myśliwa-Kurdziel

**Affiliations:** 1 Department of Biophysics, University of Life Sciences in Lublin, Akademicka 13, 20-950 Lublin, Poland; 2 Department of Agricultural, Forest and Transport Machinery, University of Life Sciences in Lublin, Głęboka 28, 20-612 Lublin, Poland; 3 Department of Analysis and Differential Equations, University of Warmia and Mazury in Olsztyn, Słoneczna 54, 10710 Olsztyn, Poland; 4 Faculty of Biochemistry, Biophysics and Biotechnology, Jagiellonian University, Gronostajowa 7, 30-387 Kraków, Poland

**Keywords:** Plant sciences, Environmental sciences

## Abstract

The aim of the study was to measure the tensile strength of 4-year-old alfalfa leaves cultivated from seeds exposed to pre-sowing stimulation with He–Ne laser light for 1 or 5 min (designated respectively as F1 and F5) and alternating magnetic field with the exposure time of 1 or 5 min (respectively, L1 and L5). The leaves were measured in terms of blade length and width as well as petiole thickness prior to the tensile test. Measurements were also conducted to determine the chlorophyll fluorescence lifetime and content of photosynthetic pigments (chlorophyll *a*, *b*, *a* + *b* and carotenoids). The observed tensile strength was between 1.59 and 2.45 N. In the test group, the observed strength was lower in leaves collected from the top and central section of the stem but higher in the bottom part of the stem as compared to the control. The maximum increase of the tearing tensile force relative to the control (C) was observed for the L1 and F1 stimulation samples in leaves collected from the top and bottom parts of the stem, while the maximum decrease for that force was recorded for L5 leaves from the middle and top part of the stem. Chlorophyll fluorescence lifetimes and the overall content of photosynthetic pigments (chlorophyll *a* and *b* and carotenoids) were noticeably decreased in the leaves subjected to the stressors/stimulants applied. The results obtained for F1, L5 and, L1 stimulation revealed a decrease in fluorescence lifetimes. The content of photosynthetic pigments was also decreased under the influence of laser light stimulation (L1). This was a clear indication of plant ageing.

## Introduction

Alfalfa, referred to as the “*queen of forage crops*”, is among the most important perennial legumes cultivated in the world^1–3^. It is a good source of natural xanthophylls and their derivatives, vitamins (including: C, D, K, E, and P), and minerals (particularly iron and copper)^4^. Above all, it is a plant used as high-protein forage in the form of soilage, hay, and silage in stock farming^1,5^. Alfalfa is also used in biofuel production^6^ and as sprouts in human nutrition^7^. It is also used in ecological agriculture as a so-called aftercrop, an excellent green fertilizer facilitating the cultivation of crops and vegetables^1^.

Photosynthetic pigments are essential for the absorption of light used as a source of energy in photosynthesis, which enables biomass production. Carotenoids additionally protect leaf tissues against photooxidative damage^8,9^. Procedures involving the measurement of chlorophyll fluorescence are gaining popularity in agriculture, horticulture, ecological farming, and seed production. The technique allows one to evaluate the physiological condition of plants through a careful analysis of the photosynthetic mechanism, and to determine whether a plant is coping with increased stress, e.g. temperatures that are too high/low, drought/excess of water, salination, etc.^10–12^.

Mechanical losses due to mowing can significantly impact the quality of alfalfa harvests. Protection of leaves against mechanical damage and separation thereof from stalks is particularly important in the context of the leaves’ digestibility by animals. Leaves are particularly vulnerable to such damage in all types of plants, and alfalfa is no exception^13^. As follows from a study by Pellizzi and Lisa^14^, such losses amount to 2–3% when press harvesting Fabaceae (with the moisture content of 28–30%).

To date, most published studies have focused on the mechanical properties of alfalfa or wheat stems, with a view to improving the design of machinery such as mowers, presses or shredders used during harvest^15,16^. Zhang et al.^17^ studied the cracking strength of tomato stems during harvest, Du and Wang^18^ carefully analyzed agricultural engineering research related to the mechanical strength of arable crops (including alfalfa, wheat, barley, rape, corn, etc.).

Precise information on such mechanical properties is valuable when designing, analyzing, and improving agricultural machinery, as well as in the context of the increasingly widespread biofuel production^18,19^. Fresh alfalfa leaves (without stems or petioles) are used in the production of a valuable juice and as additives to springtime (depending on the region) salads^20^. Given the above, we were inspired to explore the research problem related to the tensile strength of alfalfa leaves being separated from petioles. Leaves are constantly exposed to a variety of abiotic factors such as wind, precipitation, or damage caused by feeding insects and herbivores^8,21,22^. Hence, steps should be taken to avoid any additional damage, particularly when good quality of leaf blades is a priority in plants intended for consumption in salads or juices.

Global literature has relatively few papers focusing on perennial plants such as alfalfa, mainly due to the considerable effort needed to perform such studies and obtain meaningful results. This was among the reasons that encouraged us to look into this important research problem. The aim of the presented experiment was to analyze 4-year-old plants grown from seeds previously subjected to stimulation using laser light and alternating magnetic field, in terms of their mechanical strength and the general physiological condition of their photosynthetic system. Moreover, the study also aimed to determine whether parameters such as petiole thickness and leaf mass, combined with relevant photosynthetic parameters, are factors influencing the plants’ overall condition or rather, the effects of the same. Our choice of this subject matter was motivated by the fact that alfalfa leaves separated from the stems are edible, both by humans—in the form of juices, and animals—as feed pellets, while the plant’s stems are a valuable material in the production of biofuels, all of which add to the significance of these considerations.

## Materials and methods

### Plant material

The research material consisted of 4-year-old leaves of alfalfa, Ulstar cultivar, obtained from the field experiment conducted by the Department of Plant Production Technology and Commodity Science, University of Life Sciences, in Felin (51°13′21.9″ N, 22°37′55.85″ E), on a soil classified in the good wheat complex (soil quality class III a). The leaves were harvested on May 25, 2018, during the budding phase (BBCH scale 55–59).

### Electromagnetic stimulation

The leaves were grown from seeds subjected to pre-sowing treatment: C (control—unstimulated seeds); L1 and L5—seeds stimulated with laser light with 1 and 5 min exposure, respectively; and F1 and F5—seeds stimulated with a magnetic field with 1 and 5 min exposure, respectively.

**Figure 1:**
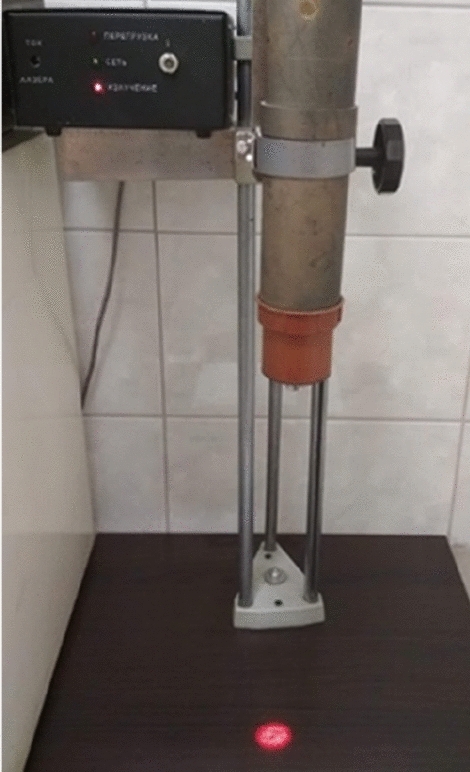
Self-designed rig for laser light stimulation of seeds. Photo by Agata Dziwulska-Hunek.

**Figure 2:**
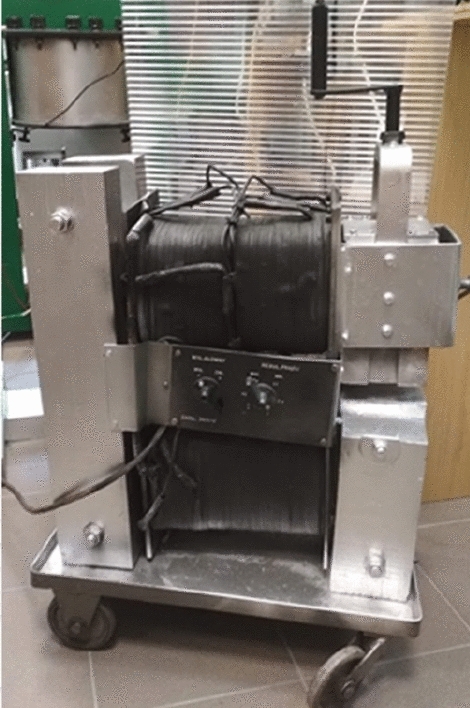
Electromagnet. Photo by Agata Dziwulska-Hunek.

Light stimulation was done using an He–Ne laser with the wavelength of 632.8 nm and surface power density of 3 mW cm^−2^. A proprietary and unique rig was created for this purpose (Fig. 1), with the divergent laser light beam directed downwards onto seeds placed in a single layer at the bottom of a dish. An electromagnet (Fig. 2)^23^ was used for the purpose of stimulating seeds with an alternating magnetic field with the magnetic induction of 30 mT.

### Mechanical strength of leaves

**Figure 3:**
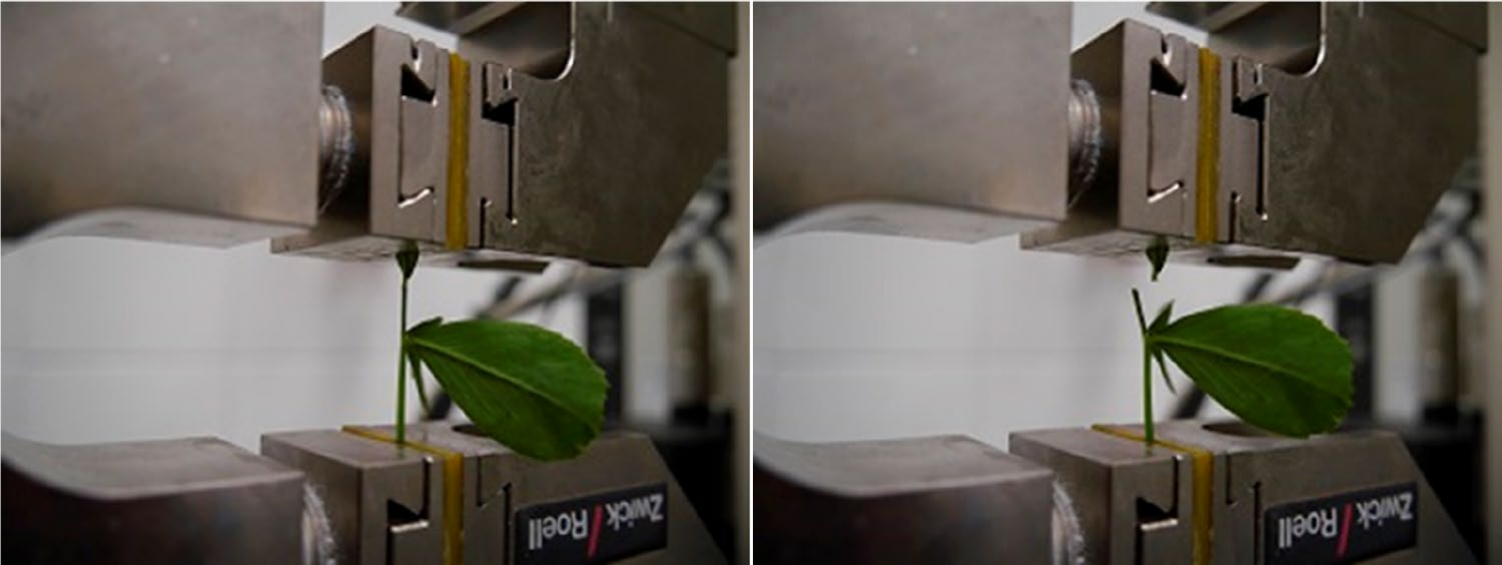
Before and after tensioning with the Zwick/Roell Z005 instrument. Photo by Agata Dziwulska-Hunek.

**Figure 4:**
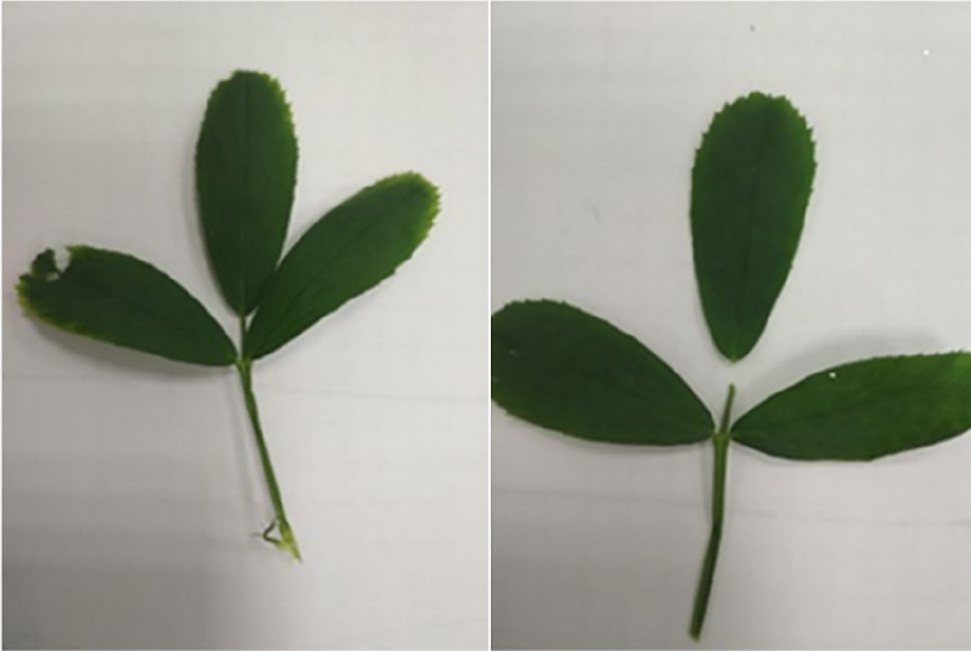
A leaf before and after tensioning. Photo by Agata Dziwulska-Hunek.

**Figure 5:**
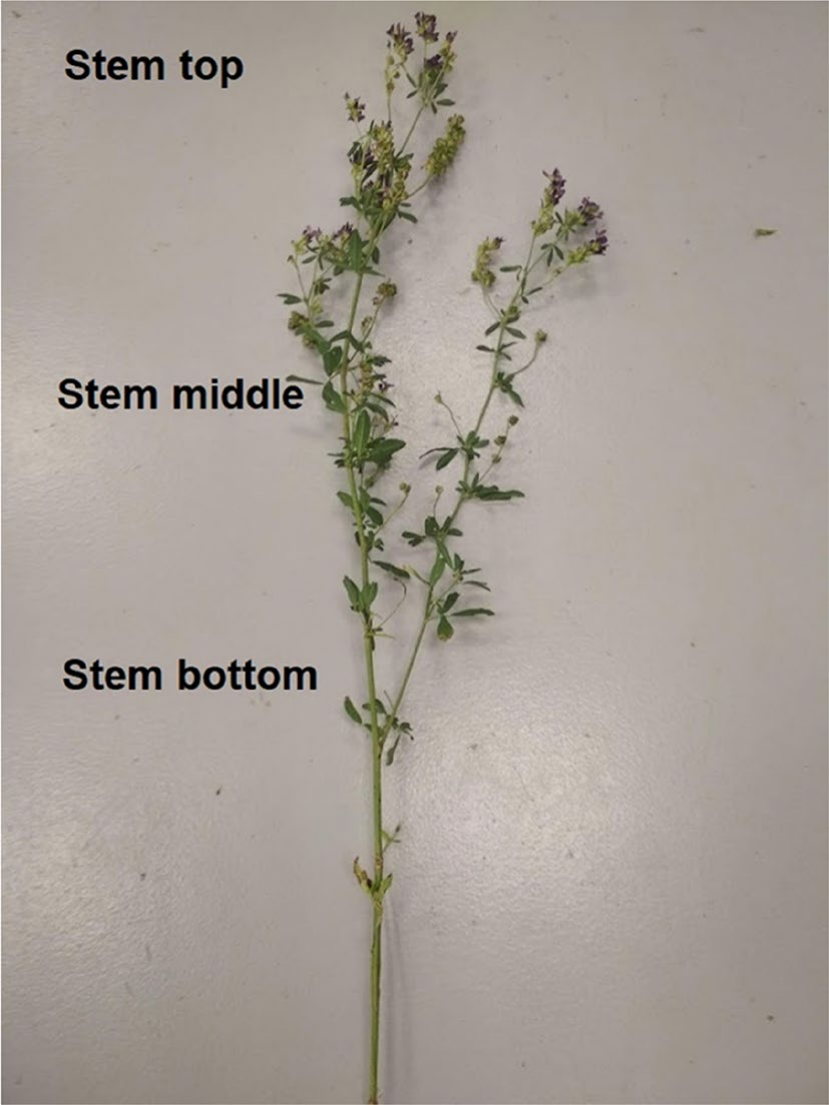
Parts of the stem from which leaves were collected for testing. Photo by Agata Dziwulska-Hunek.

The tension test was conducted unsung a Zwick/Roell Z005 instrument equipped with data registration software Test Xpert II.V3.5 by Zwick. The tensioning was done using a 50 N measurement head at the rate of 10 mm s^−1^ (Figs. 3 and 4). The temperature at the laboratory was maintained at 20 °C during the measurement. The tested leaves were collected from the top, middle, and bottom parts of plant stems (Fig. 5). Each sample measurement was repeated five times.

### Fluorescence lifetimes

Measurements of fluorescence decay were done using a K2 phase-modulation fluorimeter (ISS, USA). The leaves were crushed in a mortar in a Hepes–NaOH buffer (25 mM; pH 7.8) containing 1 mM of MgCl_2_, 1 mM of EDTA, and 0.4 M of sorbitol. The homogenate was diluted using the buffer so that the absorbance of the sample for 440 nm did not exceed 0.15. The sample was excited with 440 nm waves and the fluorescence was observed through a KV550 cut-off filter (λ > 550 nM). Measurements were done in a 1 × 1 cm cuvette, at room temperature, at 10 frequencies of exciting wave modulation (in the range of 2–200 MHz), relative to the diffusing suspension (Ludox^®^, Sigma Aldrich). Vinci2 software (ISS, USA) was used in the analysis for a double exponential and triple exponential fluorescence decay model that best corresponded to the results obtained. All measurements were done in triplicate.

### Photosynthetic pigments

Determination of the content of photosynthetic pigments. Photosynthetic pigments were isolated from leaves using acetone containing 0.01% w/v BHT (butylated hydroxytoluene), in darkness, after which the contents of chlorophyll and carotenoids were determined. The UV–Vis spectrum was measured using a Carry Bio 300 spectrometer and analyzed in accordance with the procedure published by Lichtenthaler and Buschmann^24^. Each sample was analyzed in triplicate.

### Statistical analysis

The experimental results were processed with STATISTICA 13.1 software, using the ANOVA variance analysis and post-hoc LSD test at the significance level of α = 0.05. The relations between variables were determined using Pearson’s linear correlation coefficient.

We confirm that experimental and field studies on used plants cultivated in the study, are comply with relevant institutional and national guidelines and legislation effectual at Research Centre for Cultivar Testing^25^.

## Results and discussion

### Mechanical tensile strength

Leaf parameters measured prior to the tensile strength test included the length and the width of the leaf and the thickness of the petiole, as well as the mass of leaf without the petiole measured after the test, are presented in Table 1. The tensile test measured the force needed to detach the leaf from the petiole (Table 2). In general, the seed stimulation did not significantly affect the tested parameters. Nonetheless, a certain reduction thereof was noted for the top and middle sections of the stem. On the other hand, leaves from the bottom part of the stem showed contrary results with the leaf length and petiole thickness visibly increased due to the electromagnetic field stimulation.

**Table 1:** Parameters characterizing alfalfa leaves.

Stimulation factors	Top part of the stem	Middle part of the stem	Bottom part of the stem
**Length (cm)**			
C	3.00 ± 0.49a	3.18 ± 0.62a	2.14 ± 0.29a
L1	3.20 ± 0.12a	3.22 ± 0.55a	2.30 ± 0.88a
L5	2.84 ± 0.29a	3.18 ± 0.18a	2.00 ± 0.45a
F1	2.80 ± 0.12a	2.94 ± 0.30a	2.66 ± 0.50a
F5	2.94 ± 0.43a	2.98 ± 0.51a	2.60 ± 0.27a
**Width (cm)**			
C	1.30 ± 0.32a	1.76 ± 0.32a	1.38 ± 0.51a
L1	1.32 ± 0.15a	1.64 ± 0.22a	1.59 ± 0.62a
L5	1.06 ± 0.23a	1.54 ± 0.22a	1.34 ± 0.06a
F1	1.24 ± 0.25a	1.38 ± 0.16a	1.40 ± 0.24a
F5	1.30 ± 0.34a	1.76 ± 0.69a	1.76 ± 0.47a
**Petiole thickness (mm)**			
C	0.548 ± 0.190a	0.660 ± 0.173a	0.368 ± 0.204a
L1	0.540 ± 0.073a	0.592 ± 0.198a	0.456 ± 0.247a
L5	0.432 ± 0.061a	0.656 ± 0.069a	0.364 ± 0.092a
F1	0.484 ± 0.063a	0.666 ± 0.150a	0.444 ± 0.125a
F5	0.506 ± 0.245a	0.624 ± 0.237a	0.542 ± 0.235a
**Mass of leaf without petiole (g)**			
C	0.713 ± 0.084a	0.803 ± 0.123a	0.632 ± 0.137a
L1	0.769 ± 0.034a	0.821 ± 0.096a	0.691 ± 0.193a
L5	0.743 ± 0.090a	0.790 ± 0.085a	0.651 ± 0.094a
F1	0.668 ± 0.084a	0.802 ± 0.081a	0.647 ± 0.059a
F5	0.702 ± 0.109a	0.750 ± 0.062a	0.735 ± 0.084a

*C* control (unstimulated seeds); *L1, L5* He–Ne laser light stimulation with 1 and 5 min exposure, respectively, *F1, F5* alternating magnetic field stimulation with 1 and 5 min exposure, respectively. a, b—different letters in respective columns indicate statistical differences, ± standard deviation, n = 5.

**Table 2:** Tensile force detaching the leaf from the petiole.

Stimulation factors	Top part of the stem	Middle part of the stem	Bottom part of the stem
**Force (N)**			
C	2.16 ± 0.48a	2.45 ± 0.53a	1.59 ± 0.62a
L1	2.32 ± 0.13a	2.36 ± 0.48a	1.77 ± 1.00a
L5	1.58 ± 0.20bd	2.12 ± 0.20a	1.65 ± 0.45a
F1	1.79 ± 0.25ac	2.32 ± 0.25a	2.03 ± 0.60a
F5	1.99 ± 0.42ac	2.21 ± 0.46a	1.93 ± 0.41a

*C* control (unstimulated seeds), *L1, L5* He–Ne laser light stimulation with 1 and 5 min exposure, respectively, *F1, F5* alternating magnetic field stimulation with 1 and 5 min exposure, respectively. a, b—different letters in respective columns indicate statistical differences, ± standard deviation, n = 5.

Data regarding the parameters recorded after tearing the leaves grown from seeds subjected to electromagnetic stimulation are presented in Table 2. The tensile strength of leaves was measured at approx. 1.58–2.45 N. Electromagnetic stimulation lowered the tensile strength of leaves that were collected from the top and middle parts of the stem, especially for L5 and F5. The significant decrease was observed for the L5 treatment for the top part of the stem, where the value was 73% relative to the control. A slight increase of this parameter was observed for the bottom leaves. The observed difference may be due to the dimensions of the leaves or the thickness of the stem, or possibly the overall shape of leaves. An interaction between variables L5 and F1, L5 and F5 was observed. It should be noted that the mass of leaves without petioles (from the top) was higher in the stimulated object L5, as compared to objects F1 and F5. In turn, the petiole thickness and tensile strength was reduced.

As mentioned above, the tensile force recorded in our study was between 1.58 and 2.45 N. The parameter may have been influenced by both the stimuli employed and the leaves’ enervation. The results do suggest, however, that the pre-sowing stimulation primarily affects early stages of plant growth, whereas the tensile strength as such is significantly more dependent on leaf enervation than pre-sowing stimulation. The mechanical properties of leaves are vital to their protection against damage caused by herbivores or abiotic factors (e.g.: drought, wind, precipitation, etc.)^8,26^. As follows from a study by Sahaf and Sharon^27^, mechanical stress caused by loads placed on a leaf affects its mechanical properties (elasticity modulus). The mean values of Young’s modulus were shown to change, depending on the season, for: maple 4.06–24.13 MPa and linden 1.01–16.57 MPa, and in terms of tensile forces within the range of 0.21–0.52 N mm^−1^ (maple) and 0.14–0.38 N mm^−1^ (linden)^8^. Young’s modulus values for black salsify leaves grown from seeds stimulated with laser light at various exposure times oscillated between approx. 3 and 10 MPa^28^.

In a study conducted by Kawai et al.^26^, the authors considered the influence of the physiological characteristics of leaves from various types of trees on their mechanical and structural properties, and concluded that the same were independent of each other. For the respective tree species, the leaf tensile strength ranged from 0.29 to 0.34 N mm^−1^, and Young’s modulus values for stretching were within the range of 9.68–79.9 MPa.

The tensile strength of a leaf depends on the number of veins in its blade. It has been observed that in single-vein leaves, the strength was ~ 1.07 N mm^−2^, but was significantly lower in three-vein leaves. In leaves with four veins, the tensile strength was higher^29^ at 5.91 N mm^−2^.

### Fluorescence lifetimes

Measurements of chlorophyll fluorescence lifetime provide information on the functional condition of photosynthetic complexes, particularly on the photosystem II. In the studied homogenates, we observed double or triple exponential decay of chlorophyll fluorescence (Table 3). The obtained lifetime components were within the range of 0.1–0.37 ns (t_1_), 2.04–3.61 ns (t_2_), and between 56 and 151 ns (t_3_), with the share of 45–74% (f_1_) and 20–51% (f_2_). In the samples prepared from stimulated leaves a reduction of the t_2_ component was observed, particularly for laser light stimulation (L1, L5). The reduction of fluorescence lifetimes suggests increased efficiency in other channels of chlorophyll deexcitation, including those related to energy dissipation in photosynthetic antennae. This, in turn, may signify considerably better overall results in terms of general plant growth, which is commonly observed when plant of this type, not only alfalfa, are subjected to stimulation. In the samples of leaves stimulated with the magnetic field, the third component (t_3_) was not observed in values corresponding to delayed fluorescence. Analyses of fluorescence lifetimes indicated slight functional changes in the photosynthetic complexes in leaf samples stimulated with either of the two factors, but the character of those changes was different depending on the specific factor employed.

**Table 3:** Fluorescence lifetimes (t) and corresponding fractional intensities (f) in the analyzed alfalfa leaves.

Stimulation factors	t_1_ (ns)	f_1_	t_2_ (ns)	f_2_	t_3_ (ns)	f_3_	_*χ*_^2^
C	0.31 ± 0.03	0.667	3.61 ± 0.3	0.289	151 ± 80	0.044	1.33
L1	0.29 ± 0.01	0.694	2.09 ± 0.2	0.25	56 ± 10	0.056	2.5
L5	0.30 ± 0.05	0.737	2.04 ± 0.5	0.202	184 ± 90	0.061	1.89
F1	0.10 ± 0.05	0.594	3.58 ± 0.5	0.406	–	–	12.1
F5	0.37 ± 0.013	0.448	2.99 ± 0.06	0.512	–	–	12.6

*C* control (unstimulated seeds), *L1, L5* He–Ne laser light stimulation with 1 and 5 min exposure, respectively, *F1, F5* alternating magnetic field stimulation with 1 and 5 min exposure, respectively.

Stimulation with He–Ne laser light noticeably altered the chlorophyll fluorescence lifetime in scorzonera leaves grown from a batch of 2009 seeds (4-year-old), yielding results in the ranges from 0.09 to 1.36 ns (t_1_) and from 0.89 to 1.78 ns (t_2_), with a ratio of 16–100% (f_1_) and 35–84% (f_2_). Is should be underlined that the time t_2_ was reduced in the stimulation objects for this batch of seeds^30^. This clearly suggests additional pathways of deexcitation for chlorophyll molecules present in the plants selected for study.

In literature reports, reduction of fluorescence lifetime (below 3.00 ns) has been interpreted e.g. as a sign of ageing^31^. This may be related to the age of seeds/plants in the case of perennial plants or, as in our case, to the exposure to particular stressors. Indeed, it may be due to the fact that stimulation usually allows one to obtain better quality plants faster, which in turn may necessitate earlier harvest. Otherwise, plants may be susceptible to accelerated degradation beyond a certain point. In studies analyzing extracts from young and old alfalfa plants, it was observed that laser light clearly extended fluorescence lifetimes in plants of the Radius cultivar, but reduced the same in the Ulstar cultivar (2- and 6-year-old plants), relative to the control. This may evidence a better quality of a given cultivar and its better suitability for cultivation. Moreover, fluorescence lifetimes observed in older plants are clearly shorter than those observed in young plants^32^.

### Photosynthetic pigments

Two types of chlorophyll are present in plants: *a* and *b*. Chlorophyll *a* plays an important role in the process of photosynthesis, whereas chlorophyll *b* and carotenoids are auxiliary pigments. It is noteworthy that the correct ratio of chlorophyll and carotenoids reflects the overall condition of a plant but is also noticeably dependent on the respective plant species^33^. In our study, as presented in Table 4, the content of photosynthetic pigments in alfalfa leaves decreased in plants grown from seeds subjected to electromagnetic stimulation before sowing. The most noticeable reductions were observed in study objects L1—approx. 60% Chl *a* and *b*, and F1—Chl *b* 43%). The ratio of Chl *a*/*b* was between 3.02 (F5) and 3.67 (L5). In studies conducted to date, the decrease of chlorophyll *a* and *b* concentration may have been due to electromagnetic stimulation, or the age of plants. In earlier studies, the use of laser light stimulation resulted in a decrease of chlorophyll *a* concentration in 1- and 2-year-old leaves, but an increase thereof in 3-year-old leaves^30^. In conditions of excessive hydration of alfalfa plants, Smethurst and Shabala^34^ also observed reduced chlorophyll *a* and *b* concentrations. In another study conducted by Kang et al.^35^ it was observed that chlorophyll *a* and *b* concentration decreased by approx. 45% and the concentration of carotenoids by approx. 51% in plants exposed to drought conditions, as compared to the control. The use of isolators AFFR02 and Mj1212 lessened the stressogenic effects of drought on alfalfa plants and even triggered an increase in the content of both chlorophyll by approx. 23–16% and carotenoids by approx. 55%. In studies investigating the regulator Tytanit, it was observed that it caused an increase in Chl *a* and Chl *b* content by approx. 12 and 15%, respectively, compared to the control^36^.

**Table 4:** Content of photosynthetic pigments in alfalfa leaves.

Stimulation factors	Chlorophyll *a* (µg g^−1^)	Chlorophyll *b* (µg g^−1^)	Chlorophyll *a* + *b* (µg g^−1^)	Chlorophyll* a*/*b*	Carotenoids (µg g^−1^)
C	1429 ± 314a	419 ± 77a	1848 ± 390a	3.40 ± 0.18b	183 ± 45a
L1	578 ± 19bd	164 ± 6bd	742 ± 23bd	3.52 ± 0.11b	65 ± 6b
L5	1063 ± 424a	291 ± 121a	1354 ± 545a	3.67 ± 0.06a	134 ± 82a
F1	870 ± 271b	240 ± 77bd	1110 ± 348b	3.65 ± 0.17a	127 ± 43a
F5	1134 ± 76ac	375 ± 21ac	1510 ± 97ac	3.02 ± 2.98c	68 ± 35b

*C* control (unstimulated seeds), *L1, L5* He–Ne laser light stimulation with 1 and 5 min exposure, respectively, *F1, F5* alternating magnetic field stimulation with 1 and 5 min exposure, respectively. a, b—different letters in respective columns indicate statistical differences, ± standard deviation, n = 3.

In turn, Ciupak et al.^8^ reported seasonal changes in chlorophyll *a* content in tree leaves, ranging e.g. for linden from 102.37 (fall) to 587.82 µg g^−1^ (spring), or for maple from 77.95 (fall) to 2444.5 µg g^−1^ (summer). The concentration of chlorophyll *b* ranged from 63.52 to 141.36 µg g^−1^ (linden) and from 90.89 to 897.58 µg g^−1^ (maple). In another study, Ciupak et al.^28^ studied the impact of seed stimulation with He-Ne laser light on the content of photosynthetic pigments. They observed the highest levels of chlorophyll *a* in scorzonera leaves stimulated with laser light with 1 min exposure, and the lowest with 10 min exposure, i.e. it was found that exposure time had a major impact on this parameter. Even though this seems a fairly obvious conclusion, the actual choice of correct exposure proves to be a complex problem that is further complicated by dependence on the specific type of plant as well as numerous other factors. Laser light stimulation generally triggered an increase in the pigment content in scorzonera leaves, only in L10 was a decrease by approx. 3–4% observed. This indicates that, depending on the plant species, laser light stimulation may have a positive influence on plant quality, provided that the respective are adapted to the specific needs of a given crop or vegetable.

In our study, stimulation with laser light and magnetic field triggered a decrease in carotenoid content relative to the control. This shows that electromagnetic stimulation may have constituted a stressor for the alfalfa plants selected for this study. The relevant value was within the range from 64 to 183 µg g^−1^. A significant decrease in the pigment’s content, respectively by 63 and 64%, was observed for the F5 and L1 samples. In other studies it was reported that noticeably more chlorophyll per a unit of leaf mass but less per unit of leaf surface was observed in shaded leaves, as compared to insolated leave^37^. Ibrahim and Bafeel^38^ noted a decrease in chlorophyll content and increased ratio of carotenoids to total chlorophyll in alfalfa leaves under the influence of stressogenic factors, specifically a temperature drop to 10 °C and shading for 24 h, followed by a temperature increase to 25 °C in the presence of light. In earlier studies, higher values of parameters related to photosynthesis were reported, particularly in terms of chlorophyll *a* and *b,* in legumes as compared to grass species^39^. Irshad et al.^40^ observed in their study that the content of chlorophyll in *Medicago truncatula* leaves was reduced under the influence of a stressor in the form of variable amounts of NaCl salt. Meanwhile, the He–Ne laser light treatment employed by Okla et al.^41^ facilitated the growth of *Cymbopogon proximus* plants and increased photosynthetic activity, which might also be attributed to increased chlorophyll levels in the plant. Ciupak et al.^8^ studied the content of carotenoids in linden and maple trees, and reported values within the ranges of, respectively: 147.62–168.87 µg g^−1^ and 205.55–383.99 µg g^−1^. In turn, in scorzonera leaves the observed values^28^ ranged from 107 to 135 µg g^−1^. In another study by Herde et al.^42^ investigating the impact of stress caused by light as well mechanical damage and thermal treatment of wild tomato plants, it was observed that while the stressors inhibited the process of photosynthesis, the content of chlorophyll remained stable.

**Figure 6:**
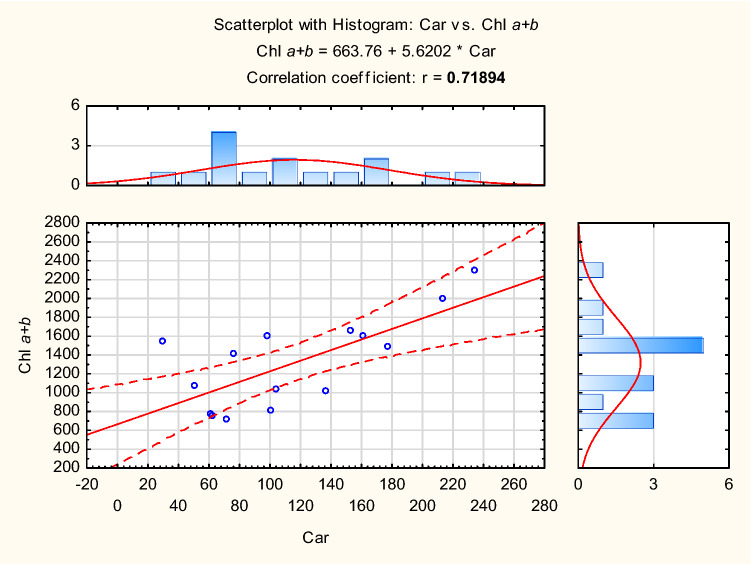
Scattering plot with histogram for correlation between carotenoids and chlorophyll *a* + *b*.

**Figure 7:**
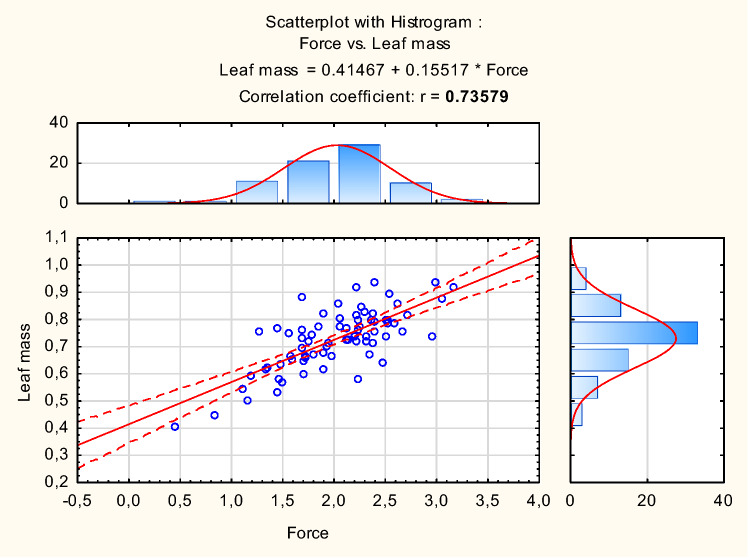
Scattering plot with histogram for correlation between disruptive force and leaf mass.

The scattering plots show two linear correlations between variables: carotenoids and chlorophyll *a* + *b* (Fig. 6), and leaf mass and tensile strength (Fig. 7). Positive values of the coefficients indicate the direction of the relation.

## Conclusions

Under the influence of the factors employed, the leaves collected from central and top parts of the plants were more fragile. Electromagnetic stimulation triggered an increase in the mass of leaves and thickness of stems in the lower parts of plants, as compared to the control. This indicated that the plants selected for the study were sturdy and resistant to tensile. This suggests that while the tensile strength may depend on the mass and thickness of leaves, the latter can also be affected by specific electromagnetic factors.

The chlorophyll fluorescence lifetime and pigment content values were noticeably reduced in the experimental alfalfa plants relative to the control, which may have been due to the electromagnetic/stressogenic factors used. This evidences ageing of alfalfa plants.

## Data Availability

All the data generated or analyzed during this study are included in this published article.
